# Trauma and health-related quality of life in patients with functional seizures

**DOI:** 10.1016/j.seizure.2025.08.009

**Published:** 2025-08-07

**Authors:** Jonah Fox, M. Junaid Humayun, Madelyn K. Bollig, Benjamin W. Hopkins, Murli Mishra

**Affiliations:** Department of Neurology, Vanderbilt University Medical Center, Nashville, TN, United States

**Keywords:** Psychogenic non-epileptic seizures, Traumatic experiences, Morbidity, Stress, Abuse

## Abstract

**Purpose:**

To assess the relationship between adverse life events and health-related quality of life in patients with functional seizures.

**Methods:**

We recruited and consented patients with functional seizures who were admitted to the epilepsy monitoring unit and asked them to complete the Life Event Checklist for DSM-5 (LEC-5) and the 36-Item Short Form Health Survey (SF-36) to evaluate adverse life events and health-related quality of life, respectively. Additional variables were collected with a retrospective chart review. A multiple linear regression model was used to evaluate independent predictors of health-related quality of life.

**Results:**

A total of 97 patients were included, and 98 % identified at least 1 adverse life event that was either witnessed or personally experienced. Health-related quality of life was poor compared to the general population. An increased number of types of witnessed or personally experienced adverse life events were associated with a worse mental component summary score and older age was associated with a worse physical component summary score. A longer duration of time with FS was associated with a better mental component summary score which may reflect some degree of adaptation or improved coping skills that developed over time.

**Conclusion:**

Our study found that an increased burden of adverse life events in patients with functional seizures impacts health-related quality of life. These findings may have treatment and resource allocation implications.

## 1. Introduction

Functional seizures (FS) are a type of functional neurological disorder (FND) characterized by transient sensory, motor, cognitive, or autonomic signs and symptoms that resemble epileptic seizures but are not caused by abnormal synchronous electrical activity in the brain [1]. Adverse life events (ALE) have been recognized as an experiential risk factor for the development of FS since at least Briquet’s 1859 Clinical and Therapeutic Treatise on Hysteria [2]. Explanatory models and recent studies continue to suggest a strong association between ALE and FS [3,4]. The prevalence of ALE in patients with FS varies significantly from study to study likely due to methodological heterogeneity [3,4]. ALE are associated with the development of health problems including obesity, cardiovascular disease, substance use disorders and other chronic conditions [5–7]. Patients with FS and ALE have been found to have a diminished quality of life and greater psychopathology compared to those with FS who did not have ALE [8].

Health-related quality of life (HRQoL) refers to an individual’s perception of their physical, mental and social well-being in relation to their health status. In the general population it has previously been demonstrated that ALE are associated with a lower HRQoL [9]. It has also been shown that those with exposure to multiple ALEs have poorer health and HRQoL compared to those with fewer or no ALEs [5,10]. It remains unclear how ALE impact HRQoL in patients with FS. We hypothesized that most patients with FS would have ALE if systematically assessed and that a greater quantity of ALE would be associated with a lower HRQoL. A better understanding of this matter could help facilitate improved patient care and may help tailor treatment strategies. Therefore, we evaluated ALE and HRQoL using validated surveys in FS patients who were admitted to the epilepsy monitoring unit.

## 2. Methods

### 2.1. Study design

This prospective study was performed at a tertiary medical center that predominantly serves patients throughout Tennessee, north Alabama, and south Kentucky in the United States. Data was collected between September 2023 and February 2025. Patients who were admitted to the epilepsy monitoring unit (EMU) for diagnostic evaluations and diagnosed with FS without coexisting epilepsy were offered to participate and consented. Survey questionnaires were subsequently completed on an electronic tablet device in RedCap [11]. The surveys had to be reformatted slightly to facilitate their use and completion on tablets in Redcap. These changes were primarily typographical in nature. Additional demographic and clinical data were retrospectively collected from the patient’s chart. This study was reviewed and approved by the Vanderbilt University Medical Center Institutional Review Board.

### 2.2. Survey questionnaires

The Life Event Checklist for DSM-5 (LEC-5) is a widely used self-report measure of ALE history that consists of questions about 17 different types of ALE [12]. The survey also assesses the degree of proximity that an individual may have had to each ALE (i.e. experienced, witnessed, learned about, or part of my job). The LEC-5 has good test-retest reliability and convergence with other similar instruments such as the Traumatic Life Events Questionnaire (TLEQ) and Clinician-Administered PTSD Scale for DSM–5 (CAPS) [12–14]. Different methods for scoring the LEC-5 have been described and studied including weighting according to the proximity of the events [15]. We focused on events that were either directly experienced or witnessed since they have been shown to have a bigger psychological impact [15, 16].

The 36-Item Short Form Health Survey (SF-36) is a validated and reliable questionnaire that may be used to assess HRQoL [17]. Responses were transformed into a single scale ranging from 0 (worst possible health status) to 100 (best possible health status) according to the instructions from the authors [18]. The SF-36 contains 9 subscales including physical functioning, role limitations due to physical health, role limitations due to emotional problems, energy/fatigue, emotional well-being, social functioning, pain and general health [19]. The SF-36 does not provide support for calculating a single total score but it is possible to calculate the total score for each of the subscales [19]. There are two higher-order summary scores that can be calculated from the subscales: the physical component summary (PCS) and mental component summary (MCS) scores [18]. The PCS measures physical health and how it impacts daily activities, whereas the MCS measures emotional and mental health as well as their impact on functioning. These scores are calculated using published regression weights derived from component factor analysis [20]. The PCS and MCS scores are transformed so that the mean is 50 and the standard deviation is 10.

### 2.3. Additional variables

The following demographic and clinical data were collected on all subjects: patient age at time of EMU admission, sex, estimated duration of FS at time of EMU admission, estimated monthly seizure frequency, antiseizure medication treatment, and if there was a family history of seizures. We also evaluated for the presence of comorbidities including obesity, smoking, substance use, and psychiatric diagnosis. In addition, we calculated a non-weighted Elixhauser Comorbidity Index score based on chart review.

### 2.4. Statistical analysis

The mean and standard deviation were calculated as measures of central tendency. Welch’s *t*-test was used to assess group differences for continuous variables. Fisher’s exact test was used to compare the proportions of males and females that reported exposure to specific types of traumas on the LEC-5. Multiple linear regression models were created to assess independent predictors of the PCS and MCS scores, respectively. All analyses were performed using GraphPad Prism 10.4.2 (GraphPad Software, San Diego, California). Results with p < 0.05 were considered statistically significant.

## 3. Results

### 3.1. Basic demographic and clinical characteristics as well as LEC-5 and SF-36 results

A total of 97 patients with EMU confirmed FS were included in the study. 3 patients did not answer at least 1 question about whether they experienced a particular type of trauma on the LEC-5 (2 patients did not answer 1 question, 1 patient did not answer 2 questions). 3 separate patients did not answer questions regarding the worst type of trauma that they experienced. For the purposes of this study, we assumed that these patients did not experience the type of trauma in question but otherwise included their data in the analysis. 4 patients did not complete the SF-36 (3 patients did not answer any questions from it, and 1 patient only answered the first 6 questions). We excluded these patients from analyses which used data from the SF-36 but otherwise included them.

The mean age at the time of the EMU admission was 41.08 years and 82.47 % were female (Table 1). Patients had FS for a mean of 8.11 years at the time of admission and 82.47 % were taking anti-seizure medications. Most patients had a small number of comorbidities as indicated by the mean Elixhauser summary score of 2.86 and standard deviation of 1.70.

All but two patients had at least one traumatic experience identified by the LEC-5 that either happened directly to the patient or was witnessed (Table 2). The average number of traumatic experiences was 6.02 per patient. The average time from the earliest most severe traumatic experience to the onset of FS was 14.77 years. It should be noted that 15 patients were excluded from this calculation because these patients reported that their most severe trauma occurred after the onset of FS. The worst trauma involved someone’s life being in danger in 62.77 % and it involved someone being seriously injured or killed in 48.94 %. Sexual violence was involved in the worst trauma for 27.66 % of patients. Information regarding the proportion of patients that directly experienced or witnessed each type of trauma is detailed in Table 3. Differences in the types of traumas according to sex can be found in the supplementary table. Women were significantly more likely to experience or witness a sexual assault and other unwanted or uncomfortable sexual experiences. Men were significantly more likely to experience or witness exposure to a toxic substance, a fire or explosion, as well as combat or exposure to a warzone.

### 3.2. Multiple linear regression

Patients with FS had significantly worse scores on all subscales and both higher-order summary scores when compared to data from the general United States population (Table 4) [18]. The multivariate analysis showed that the number of types of traumas identified on the LEC-5 was independently associated with a lower MCS score (p < 0.001, 95 % CI [−2.594, −0.724]). The duration of time that patients had FS was independently associated with a higher MCS score (p = 0.049, 95 % CI [0.001, 0.497]) (Table 5). Age at the time of EMU admission was independently associated with a lower PCS score (p = 0.005, 95 % CI [−0.426, −0.080]) (Table 6).

## 4. Discussion

This study used validated instruments to systematically evaluate ALE and HRQoL in patients with FS. We found that nearly 98 % of patients identified an ALE that was either witnessed or personally experienced. Almost half of patients reported that their worst ALE involved someone being seriously injured or killed and a little over a quarter of patients reported that their worst ALE involved sexual violence. Certain types of ALE were overrepresented according to patient sex. Overall, patients with FS had a worse HRQoL compared to a group of historic healthy controls from the general United States population [18]. A greater number of ALE were independently associated with a worse MCS score and a longer duration of time with FS was independently associated with a better MCS score. Younger patients were associated with a lower PCS score.

Previous studies have suggested that ALE can have a dose-dependent effect on health with resultant changes in the nervous, endocrine and immune systems that are further exacerbated by an increased tendency to adopt maladaptive and risky behaviors [21–24]. This may partly account for the elevated burden of comorbidities found in patients with FS which has been shown to explain their elevated mortality risk [25, 26]. An increased burden of ALE has also been shown to be associated with a lower HRQoL in the general population and amongst those with post-traumatic stress disorder [6,27]. This is important because it reflects how ALE can impact a person’s physical, mental, and social well-being. In our study, we found that among patients with FS a greater number of ALE were found to be associated with a lower MCS but not the PCS score. This may be because that although ALE may impact HRQoL in general there may be a more severe impact on emotional well-being, social functioning, and mental health perception which is reflected in the MCS score. It may be that there is a ceiling effect such that patients with FS already have limitations in their physical functioning, role limitations and bodily pain that are not further worsened by a greater number of ALE. A prior study that assessed ALE using different instruments found that it was not associated with HRQoL in the multivariate analysis [28]. This may have been because they included assessments for depression, anxiety, PTSD and alexithymia which we did not control for. Consistent with our results, however, another group previously reported that FS patients with traumatic experiences had a worse HRQoL than those who did not even when controlling for depression and anxiety [8]. The authors of the former study suggested that this discrepancy may have been because HRQoL is more likely to be negatively affected by the development of PTSD and dissociation than the mere exposure to ALE. Though we did not control for these factors, our study suggested that it may be the quantity of ALE that influences HRQoL similar to what has been found in other patient groups [6,27]. Future studies including all of these variables will need to be conducted to further clarify this matter.

To our surprise, a longer duration of time with FS was associated with a better MCS score. A prior study which evaluated the long-term course of patients with FS also found that HRQoL tended to improve over time [29]. This was attributed to a reduction in seizures over time and administration of appropriate treatments such as psychotherapy. However, in our study patients presented to the EMU for diagnosis and most were therefore less likely to be receiving treatment for FS. The onset of FS can result in a cascade of new psychological, social, and financial stressors that may be particularly intense earlier on given the significant and rapid changes which may occur. For example, patients may abruptly lose their independence, ability to drive, employment, as well as face stigmatization. Previous studies have suggested that among patients with chronic medical conditions including epilepsy that the associated severity of disability and loss of psychological resources has a greater impact on psychological distress than the characteristics of the condition itself [30,31]. This is likely also true for FS given that it can lead to disabilities similar to epilepsy. It may be that some degree of adaptation, improved coping and resilience develops over time that leads to an improved MCS score. Patients with a longer duration of FS may have been more likely to be receiving psychotherapy or other treatments for associated conditions which resulted in an improved MCS score though this was not assessed. It is also possible that some patients were already diagnosed with FS and receiving psychotherapy for it but came to the EMU to characterize new event types or to confirm the diagnosis. The potential impact of unmeasured confounders such as psychotherapy, psychotropic medications and social support from friends and family may have also influenced these findings.

A greater age at the time of EMU admission was associated with a poorer PCS score. Similar findings were found in a prior study which evaluated HRQoL in patients with FS [28]. This is not surprising given that generally as people age, they tend to accumulate physical health problems that may impact daily activities.

We found that certain types of ALE were overrepresented according to patient sex. A prior meta-analysis and systematic review of ALE and FND also reported similar findings [32]. Most patients reported several types ALE with the average being around 6 per patient. It was also found that in 84.54 % of patients that the worst ALE occurred prior to onset of FS and that among these patients it occurred an average of 14.77 years prior. Previous studies have suggested that stressful experiences tended to occur more proximally to onset of FND symptoms but we could not identify a prior study that evaluated the timing of the worst ALE [32,33]. In our clinical experience, many patients report a severe ALE that occurred years prior to the onset of FS and also a more proximal acute stressor or ALE which seemed to preciptate the onset of FS. However, it is not known exactly how the proximty, severity or type of ALE differentially influences the development or perpetuation of FS. A free text portion of survey which allowed for patients to describe their ALE in further detail revealed that some patients experienced ALE repetitively over a prolonged period of time such as in some cases of sexual abuse or exposure to war. Whereas other patients reported ALE that were more restricted in time. It is not known how these differences impacted a particular patient’s propensity to develop FS but mulitple explanatory models for FS suggest various ways that these factors may be relevant [34].

Limitations of this study include the absence of systematic evaluations for psychiatric comorbidities using diagnostic interviews, and that some of the data was collected with retrospective chart review. Given that we did not perform psychiatric assessments it limited our ability to control for comorbidities beyond what was captured by chart review in the Elixhauser Comorbidity Index score. In addition, the healthy sample from the United States that we used to compare our patient’s SF-36 scores to were not age- or sex-matched because this was not possible. It might be that our findings could partially be explained by the demographic differences between the groups. Strengths of the study include the prospective data collection using validated instruments for the assessment of ALE and HRQoL as well as the fact that all patients had EMU confirmed FS. This study adds to our understanding of the relationship between ALE and HRQoL in patinets with FS. Patients with FS may benefit from screening for ALE and appropriate referrals to trauma-based treatemtns [8].

## 5. Conclusion

This study found that almost all patients with FS had an ALE that was either witnessed or personally experienced and that HRQoL was poor. Exposure to a greater number of ALE was associated with a worse MCS and older age was associated with a worse PCS. A longer duration of time with FS was associated with a better MCS which may have reflected some degree of adaptation after living with FS for multiple years. Future studies could attempt to evaluate how treatment of FS may improve HRQoL in relationship to ALE burden and whether treatment need to be tailored according to a patient’s ALE history.

## Supplementary Material

1

Supplementary material associated with this article can be found, in the online version, at doi:10.1016/j.seizure.2025.08.009.

## Figures and Tables

**Table 1:** Demographic and clinical characteristics of patients with functional seizures.

	*N* = 97
Patient age in years at time of EMU admission, mean ± SD	41.08 ± 13.57
Female ( %)	80 (82.47 %)
Duration of FS in years at time of EMU admission, mean ± SD	8.11 ± 11.35
Estimated monthly seizure frequency, mean ± SD	21.05 ± 30.85
Taking anti-seizure medication at time of EMU admission ( %)	80 (82.47 %)
History of reported head injury ( %)	39 (40.21 %)
Reported family history of seizures ( %)	21 (21.65 %)
Obesity ( %)	61 (62.89 %)
History of smoking ( %)	36 (37.11 %)
History of substance use ( %)	19 (19.59 %)
Psychiatric diagnoses ( %)	87 (89.69 %)
Summary Elixhauser Comorbidity Index score, mean ± SD	2.86 ± 1.70

EMU: epilepsy monitoring unit; FS: functional seizures; BMI: body mass index

**Table 2:** Summative characteristics of traumas in patients with functional seizures.

	*N* = 97
At least 1 LEC-5 identified trauma that either happened directly to patient or was witnessed ( %)	95 (97.9 %)
Total number of identified types of traumas that happened to the patient or was witnessed, mean ± SD	6.02 ± 3.18
Mean time from the earliest most severe trauma to onset of FS in years, mean ± SD*	14.77 ± 13.60
Number of patients with worst trauma involving someone’s life being in danger^^^	59 (62.77 %)
Number of patients with worst trauma involving someone being serious injured or killed^^^	46 (48.94 %)
Number of patients with worst trauma involving sexual violence^^^	26 (27.66 %)

* Excludes 15 patients where the most severe trauma was after onset of seizures and 9 with missing data

^ Excludes 3 patients who did not complete the later portions of the survey

LEC-5: Life Events Checklist for DSM-5

**Table 3:** Characteristics of traumas in patients with functional seizures.

Type of trauma	Prevalence ( %) *N* = 97	Type of trauma	Prevalence ( %) *N* = 97
Number of patients that either witnessed or experienced a natural disaster*	36 (37.50 %)	Number of patients that either witnessed or experienced combat or exposure to a warzone	4 (4.12 %)
Number of patients that either witnessed or experienced a fire or explosion	24 (24.74 %)	Number of patients that either witnessed or experienced captivity (for example, being kidnapped or held hostage)	8 (8.25 %)
Number of patients that either witnessed or experienced a transportation accident	63 (64.95 %)	Number of patients that either witnessed or experienced a life-threatening illness or injury	41 (42.27 %)
Number of patients that either witnessed or experienced a serious accident at work, home, or during recreational activity	31 (31.96 %)	Number of patients that either witnessed or experienced severe human suffering	25 (25.77 %)
Number of patients that either witnessed or experienced an exposure to a toxic substance*	8 (8.33 %)	Number of patients that either witnessed or experienced sudden violent death	25 (25.77 %)
Number of patients that either witnessed or experienced a physical assault	66 (68.04 %)	Number of patients that either witnessed or experienced sudden accidental death	33 (34.02 %)
Number of patients that either witnessed or experienced an assault with a weapon*	33 (34.38 %)	Number of patients that either witnessed or experienced serious injury, harm, or death you caused to someone else	8 (8.25 %)
Number of patients that either witnessed or experienced a sexual assault	59 (60.82 %)	Number of patients that either witnessed or experienced any other very stressful event or experience*	69 (71.88 %)
Number of patients that either witnessed or experienced other unwanted or uncomfortable sexual experience	56 (57.73 %)		

* Excludes 1 patient who did not answer the question

**Table 4:** 36-Item Short Form Health Survey (SF-36) domain scores for patients with functional seizures compared to a sample from the general population.

SF-36 domains	Mean for all patients* N = 93	Mean scores for the general United States population [18]	p-value (*t*-test with Welch’s correction)
Physical functioning	50.27 ± 28.20	84.15 ± 23.28	<0.001
Role limitations due to physical health	21.77 ± 34.03	80.96 ± 34.00	<0.001
Role limitations due to emotional problems	28.67 ± 37.94	75.15 ± 23.69	<0.001
Energy/fatigue	23.92 ± 17.61	60.86 ± 20.96	<0.001
Emotional well-being	48.99 ± 20.90	74.74 ± 18.05	<0.001
Social functioning	39.92 ± 29.21	83.28 ± 22.69	<0.001
Pain	40.99 ± 25.74	75.15 ± 23.69	<0.001
General health	41.29 ± 41.29	71.95 ± 20.34	<0.001
Health change	40.86 ± 29.90	Not available	
Physical Component Summary^^^	35.93 ± 10.34	50.00 ± 10.00	<0.001
Mental Component Summary^^^	32.40 ± 13.63	50.00 ± 10.00	<0.001

* Excludes 4 patients who did not complete the SF-36

^ These are standardized scores

**Table 5:** Multivariate analysis evaluating predictors of the Mental Component Summary score.

	p-values	Beta	95 % confidence interval
Age at time of EMU admission	0.135	0.17	−0.053 to 0.386
Sex	0.163	−4.61	−11.110 to 1.896
Elixhauser summary score	0.564	−0.51	−2.265 to 1.243
Duration of time patient had functional seizures	**0.049**	0.25	0.001 to 0.497
Estimated seizure frequency per month	0.663	−0.02	−0.128 to 0.082
Number of types of traumas identified on the LEC-5	<**0.001**	−1.66	−2.594 to −0.724
Trauma involving either life in danger, serious injury or death	0.308	3.10	−2.912 to 9.117
Trauma involving sexual violence	0.685	−1.30	−7.643 to 5.043

EMU: epilepsy monitoring unit

LEC-5: Life Event Checklist for DSM-5

Each predictor’s effect is adjusted for all other covariates in the model. The 4 patients who did not complete the SF-36 were excluded from this analysis.

**Table 6:** Multivariate analysis evaluating predictors of the Physical Component Summary score.

	p-values	Beta	95 % confidence interval
Age at time of EMU admission	**0.005**	−0.25	−0.426 to −0.080
Sex	0.432	2.04	−3.093 to 7.174
Number of Elixhauser comorbidities	0.457	−0.52	−1.905 to 0.864
Duration of time patient had functional seizures	0.344	0.09	−0.102 to 0.289
Estimated seizure frequency per month	0.178	−0.06	−0.139 to 0.026
Number of types of traumas identified on the LEC-5	0.658	−0.16	−0.903 to 0.573
Trauma involving either life in danger, serious injury or death	0.804	0.59	−4.154 to 5.341
Trauma involving sexual violence	0.815	−0.59	−5.599 to 4.414

EMU: epilepsy monitoring unit

LEC-5: Life Event Checklist for DSM-5

Each predictor’s effect is adjusted for all other covariates in the model. The 4 patients who did not complete the SF-36 were excluded from this analysis.
