# Supplementary material for: Trauma and health-related quality of life in patients with functional seizures
Source: Seizure. Author manuscript; Available in PMC 2026 Jun 5. (PMC13237828; doi:10.1016/j.seizure.2025.08.009)
Supplement: 1 [file NIHMS2180960-supplement-1.docx]

Supplementary table 1: Characteristics of traumas in patients with functional seizures according to sex.

| Type of trauma | Prevalence (%) N=97 | Type of trauma | Prevalence (%) N=97 |
| --- | --- | --- | --- |
| Number of patients that either witnessed or experienced a natural disaster* | Male: 8/16 (50.00%) Female: 28/80 (35.00%)  p-value: 0.272 Odds Ratio (95% CI): 1.857 (0.590-5.840) | Number of patients that either witnessed or experienced combat or exposure to a warzone | Male: 4/16 (25.00%) Female: 0/81 (0.00%)  **p-value: <0.001 Odds Ratio (95% CI): Undefined (–)^** |
| Number of patients that either witnessed or experienced a fire or explosion | Male: 9/16 (56.25%) Female: 15/81 (18.52%)  p-value: **0.003** Odds Ratio (95% CI): **5.657 (1.738-16.780)** | Number of patients that either witnessed or experienced captivity (for example, being kidnapped or held hostage) | Male: 0/16 (0.00%) Female: 8/81 (9.88%)  p-value: 0.3456 Odds Ratio (95% CI): Undefined (0)^ |
| Number of patients that either witnessed or experienced a transportation accident | Male: 10/16 (62.50%) Female: 53/81 (65.43%)  p-value: >0.999 Odds Ratio (95% CI): 0.881 (0.299-2.788) | Number of patients that either witnessed or experienced a life-threatening illness or injury | Male: 5/16 (31.25%) Female: 36/81 (44.44%)  p-value: 0.412 Odds Ratio (95% CI): 0.568 (0.205-1.684) |
| Number of patients that either witnessed or experienced a serious accident at work, home, or during recreational activity | Male: 8/16 (50.00%) Female: 23/81 (28.40%)  p-value: 0.140 Odds Ratio (95% CI): 2.522 (0.785-7.268) | Number of patients that either witnessed or experienced severe human suffering | Male: 7/16 (43.75%) Female: 18/81 (22.22%)  p-value: 0.114 Odds Ratio (95% CI): 2.722 (0.9436-8.639) |
| Number of patients that either witnessed or experienced an exposure to a toxic substance* | Male: 4/16 (25.00%) Female: 4/80 (5.00%)  **p-value: 0.025 Odds Ratio (95% CI): 6.333 (1.619-23.480)** | Number of patients that either witnessed or experienced sudden violent death | Male: 5/16 (31.25%) Female: 20/81 (24.69%)  p-value: 0.549 Odds Ratio (95% CI): 1.386 (0.481-4.361) |
| Number of patients that either witnessed or experienced a physical assault | Male: 10/16 (62.50%) Female: 56/81 (69.14%)  p-value: 0.770 Odds Ratio (95% CI): 0.7440 (0.250-2.380) | Number of patients that either witnessed or experienced sudden accidental death | Male: 7/16 (43.75%) Female: 26/81 (32.10%)  p-value: 0.397 Odds Ratio (95% CI): 1.645 (0.596-4.935) |
| Number of patients that either witnessed or experienced an assault with a weapon* | Male: 8/16 (50.00%) Female: 25/80 (31.25%)  p-value: 0.162 Odds Ratio (95% CI): 2.200 (0.691 to 6.460) | Number of patients that either witnessed or experienced serious injury, harm, or death you caused to someone else | Male: 2/16 (12.50%) Female: 6/81 (7.41%)  p-value: 0.615 Odds Ratio (95% CI): 1.786 (0.337-8.408) |
| Number of patients that either witnessed or experienced a sexual assault | Male: 3/16 (18.75%) Female: 56/81 (69.14%)  **p-value: <0.001 Odds Ratio (95% CI): 0.103 (0.030 to 0.374)** | Number of patients that either witnessed or experienced any other very stressful event or experience* | Male: 9/16 (56.25%) Female: 60/80 (75.00%)  p-value: 0.139 Odds Ratio (95% CI): 0.429 (0.138-1.219) |
| Number of patients that either witnessed or experienced other unwanted or uncomfortable sexual experience | Male: 3/16 (18.75%) Female: 53/81 (65.43%)  **p-value: <0.001 Odds Ratio (95% CI): 0.1219 (0.035-0.437)** |  |  |

*Excludes 1 patient who did not answer the question
^ Odds ratio undefined due to zero count in one cell (no females or males reported exposure).
